# A resected case of hepato-pancreaticoduodenectomy for widely extended cholangiocarcinoma undergoing previous intra-abdominal poly-surgery

**DOI:** 10.1016/j.ijscr.2018.10.035

**Published:** 2018-10-26

**Authors:** Atsushi Nanashima, Naoya Imamura, Masahide Hiyoshi, Koichi Yano, Takeomi Hamada, Teru Chiyotanda, Kenzo Nagatomo, Rouko Hamada, Hiroshi Ito

**Affiliations:** a Division of Hepato-biliary-pancreas and Digestive Surgery, Kiyotake 5200, Miyazaki, 889-1692, Japan; b Division of Plastic and Reconstructive Surgery, Department of Surgery, University of Miyazaki Faculty of Medicine, Kiyotake 5200, Miyazaki, 889-1692, Japan

**Keywords:** Hepato-pancreaticoduodenectomy, Previous poly-surgery, Careful managements

## Abstract

Introduction: This case report describes a successful radical operation for a patient with extensive advanced cholangiocarcinoma who had previously undergone intra-abdominal poly-surgery for advanced gall bladder carcinoma. Careful diagnosis to define the adequate division of the right hepatic duct was performed, and the operation was completed without postoperative complications.

Case presentation: A 61-year-old woman was admitted to a hospital for obstructive jaundice, and extra-hepatic cholangiocarcinoma was found. Seven years prior, she underwent poly-surgery, which included cholecystectomy, gastrectomy, and colectomy, for advanced gall bladder carcinoma. Although she did not receive adjuvant chemotherapy, she had no tumor relapse. She was recommended chemo-radiation therapy to treat the cholangiocarcinoma; however, she visited our hospital to inquire the possibility of receiving radical operation. Enhanced computed tomography showed extensive cholangiocarcinoma without distant metastases, which was confirmed by endoscopic biopsy. Since the transected bile duct was without cancer-invasion, which was confirmed by a negative biopsy result, we were able to perform radical left hepatectomy and pancreaticoduodenectomy (HPD). The patient was discharged without any complications. Careful preoperative examination allowed for a complex operation to be successfully completed.

Discussion: Complex surgery for advanced hepato-biliary-pancreatic malignancies after poly-surgery is difficult and requires expertise and intensive postoperative care.

Conclusion: HPB surgeons should adopt an aggressive policy to treat patients who have undergone previous major abdominal surgery.

## Highlights

• Patients undergoing poly surgery have been increased due to improvement of postoperative managements.
• We experienced a patient with widely spreading cholangiocarcinoma who had previously undergone polysurgery.
• Even though the complicated prior surgery in the abdomen, the R0 operation can be safely completed by expert surgeons.

## 1 Introduction

Radical (R0) operation is only a curative treatment option for biliary carcinomas [1,2], and invasive operations are necessary in order to not expose the tumor burden at the dissected plane. For previous poly-surgeries of the abdomen, proper diagnosis and operative planning is difficult but necessary for patient recovery and safety [3]. Particularly for biliary surgery, advanced skills and preparations for intra-operative injuries are necessary. Appropriate imaging and preoperative simulations are also important to identify various hepatic vasculatures that may be encountered during the operation [4,5].

This case report demonstrates a successful R0 hepato-pancreaticoduodenectomy (HPD) for extensive extra-hepatic cholangiocarcinoma (EC) owing to proper preoperative and intraoperative managements. This work has been reported in accordance with the SCARE criteria and cite the following paper in your references:

Agha RA, Fowler AJ, Saetta A, Barai I, Rajmohan S, Orgill DP, for the SCARE Group. The SCARE Statement: Consensus-based surgical case report guidelines. International Journal of Surgery 2016 [6].

## 2 Case presentation

**Fig. 1:**
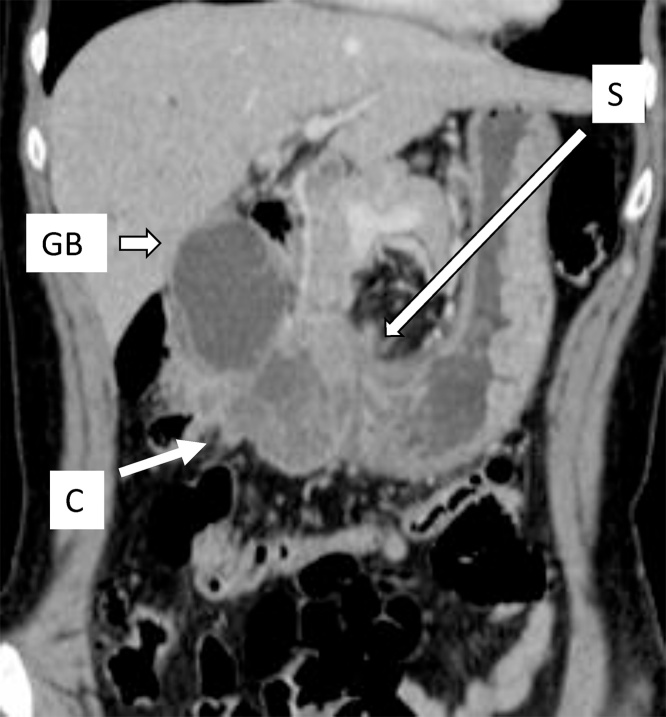
Enhanced CT taken 7 years prior showed the gall bladder (GB) carcinoma (arrow) involving the stomach (S) and right side transverse colon (C), which was resected en bloc.

A 61-year-old woman was admitted to a hospital due to obstructive jaundice. Extensive EC was found by diagnostic imaging, and she was subsequently scheduled for brachytherapy since the tumor was found to be unresectable. Seven years prior at the same hospital, she underwent multi-organ en bloc resection for advanced gall bladder (GB) carcinoma involving the distal stomach and right side transverse colon (Fig. 1). Extended cholecystectomy, distal gastrectomy, and right hemi-colectomy with loco-regional lymphadenectomy were also performed. Despite not receiving adjuvant chemotherapy, she had remained without tumor relapse. She was referred to our institute for a second opinion.

**Fig. 2:**
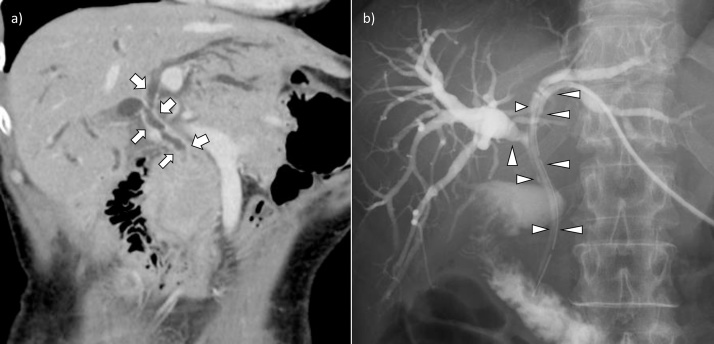
Enhanced CT showing extensive cholangiocarcinoma with wall thickening and biliary stenosis (arrow) (a). PTBD cholangiography showed irregularity at the same lesion (arrowhead) (b).

**Fig. 3:**
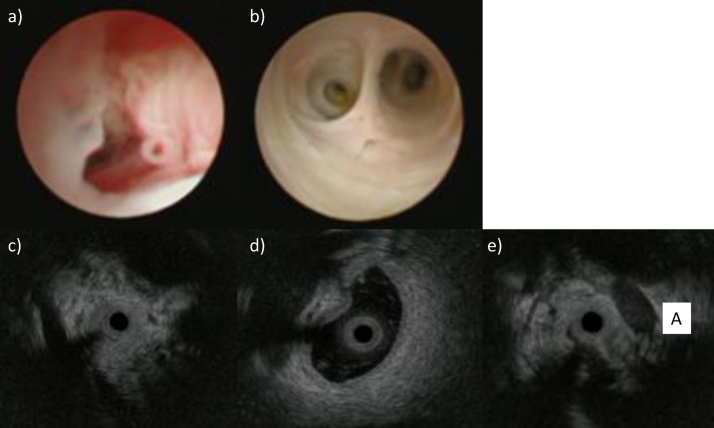
Cholangioscopy showed a narrow, papillary tumor at the stenotic lesion (a). Intra-luminal view showed that the confluence of the anterior and posterior sectorial bile duct was normal. (b). IDUS showed wall thickness at the main mass lesion (c), a thinner wall with dilatation at the confluence of the anterior and posterior sectorial bile duct (d) with no involvement of the RHA (A) (e).

Obstructive jaundice was resolved by percutaneous transhepatic biliary drainage (PTBD) via the left lateral sector of the liver at the previous hospital. Enhanced abdominal computed tomography (CT) and PTBD cholangiography showed wide stenosis of the intra- and extra-hepatic bile duct (Fig. 2a and b). Cholangioscopy and intraductal ultrasonography (IDUS) showed a papillary tumor with wall thickness and stenosis with no involvement of the adjacent right hepatic artery (RHA) (Fig. 3a and b). Endoscopic biopsy performed at the stenotic lesion and non-stenotic bile duct at the confluence of the anterior and posterior sectional branches was negative. Although invasive adenocarcinoma was diagnosed, cancer infiltration was not observed at the non-stenotic bile duct epithelium. Since neither distant nor node metastasis was observed and liver function reserve was sufficient for hemi-hepatectomy, we planned for HPD despite the possibility of tumor recurrence from GB cancer.

**Fig. 4:**
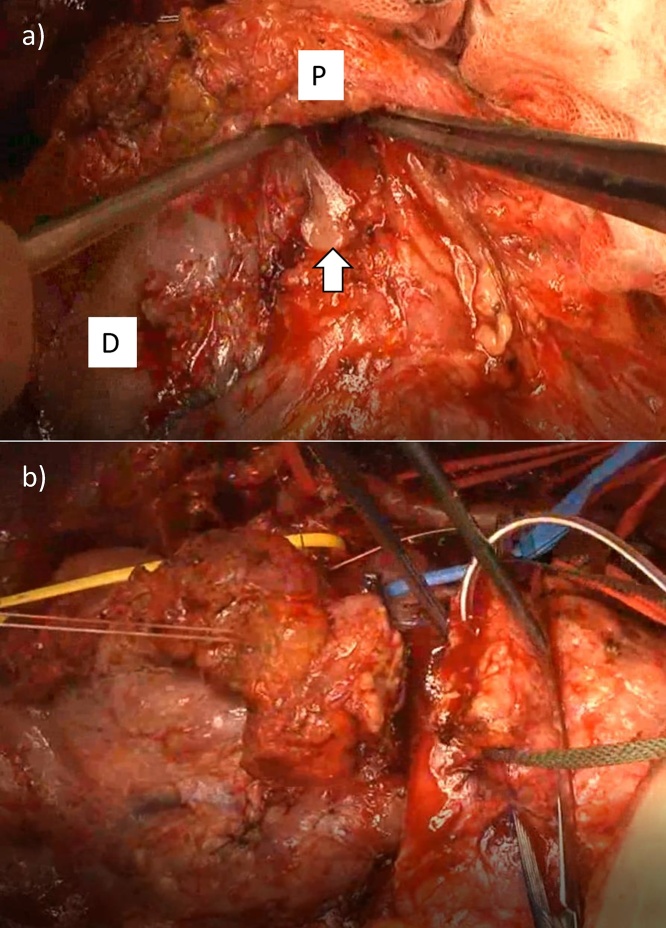
The origin of PD was placed in front of the SMV (arrow) (a), and PD was completed first (b). P, pancreas burden between head and body; D, duodenum.

**Fig. 5:**
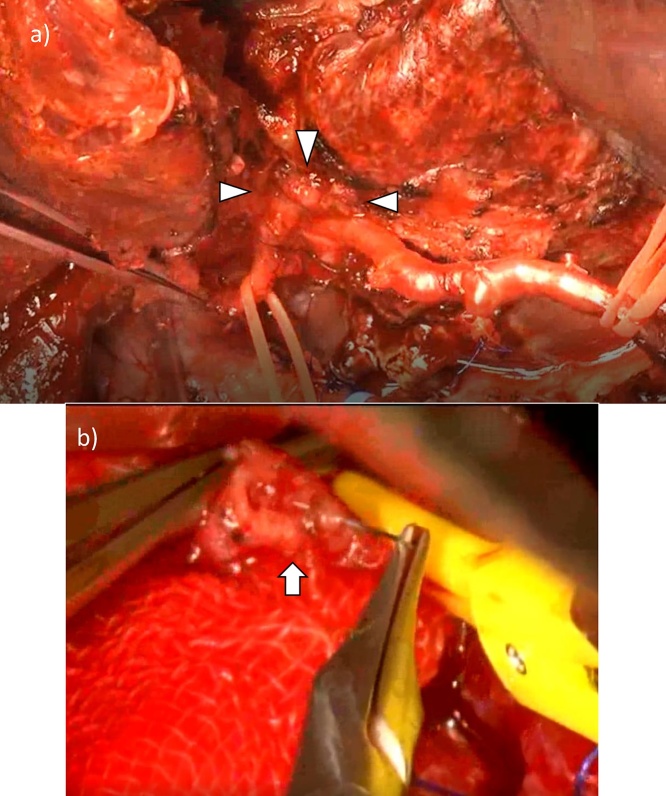
Severe adhesion between the hilar bile duct and RHA was observed (arrowhead) (a), and the anterior sectorial branch was incidentally injured and repaired by micro-surgery (arrow) (b). aRHA, anterior branch of RHA; pRHA, posterior branch.

**Fig. 6:**
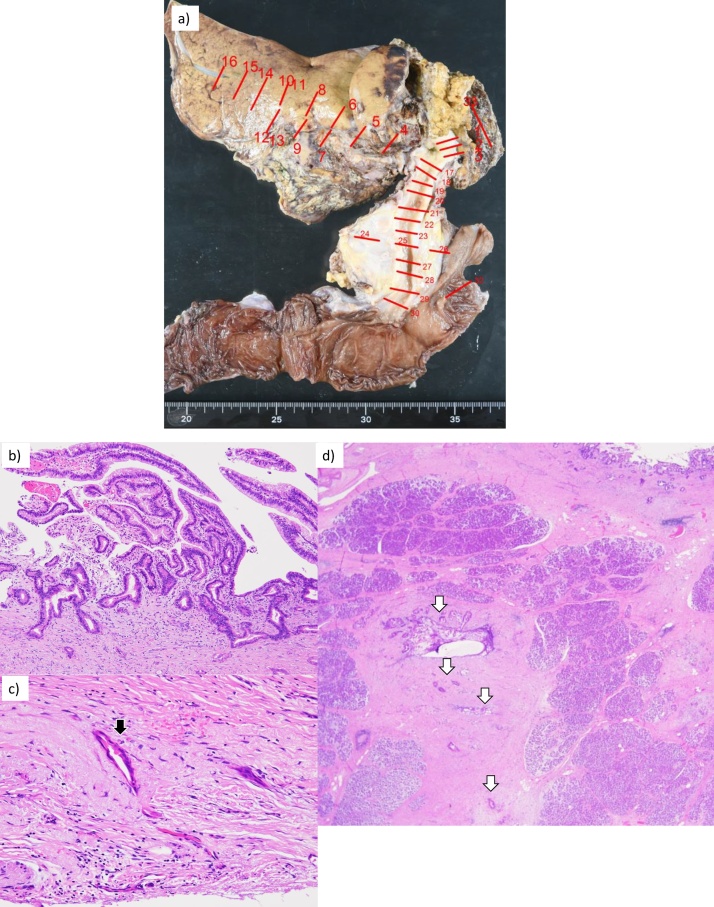
Macroscopic findings by HPD showed no remnant cholangiocarcinoma at the transected bile duct and dissected surface. a) Cholangiocarcinoma, b) papillary adenocarcinoma, c) subserosal infiltration of the cancer nest, and d) pancreatic infiltration of the cancer nest.

There was no peritoneal dissemination, liver metastasis, or distant node metastasis by laparotomy. Although there was a postoperative adhesion in the upper abdomen, the front of a superior mesenteric vein (SMV) was found and pancreaticoduodenectomy (PD) was performed (Fig. 4a and b). During exfoliation of the hilar bile duct, adhesion to the surrounding main vessels became severe, particularly in the neighboring RHA (Fig. 5a); however, this was considered non-tumor invasion by macroscopic findings. The anterior sectorial arterial branch was partially injured and was repaired with 8-0 polypropylene sutures (Fig. 5b). After anastomosis, arterial flow increased compared with dissection. Left hepatectomy with transection of the right hepatic duct was also performed at the lesion. Thus, R0 resection was performed without tumor exposure at the dissected plane (Fig. 6). Pancreatojejunostomy, hepaticojejunostomy, and jejuno-jejunostomy were also performed. The total operating time was 685 min (including 45 min for arterial repair), and blood loss was 1200 mL, which did not require blood transfusion.

Microscopic findings showed papillary adenocarcinoma with stromal and pancreatic invasions that did not extend to the dissected surface. Lymph node metastasis was found on the pancreatic surface. R0 resection was also histologically confirmed. The postoperative course was uneventful without severe damage to the liver, and the patient was discharged at day 14. Four months after HPD, she remains without tumor recurrence or complications.

## 3 Discussion

Recently, major hepatectomies and pancreatectomies have been safely performed after proper preoperative evaluation of the functional liver reserve and improvements in postoperative management [3,7]. Patient prognosis has improved with increased survival; however, findings of additional malignancies require surgical treatment. A history of prior abdominal surgeries may influence subsequent abdominal surgeries. In particular, hepatic and pancreatic resections are relatively complex surgeries and are invasive compared with other digestive tract surgeries. Adverse complications from these operations may to lead to systemic damage or lethal conditions. We previously examined (submitted but not yet published) reports of 28 patients who underwent planned hepatic or pancreatic resections after abdominal surgeries. This study demonstrates good outcomes and surgical safety for both hepatectomy and pancreatectomy in patients with prior abdominal surgery. In our case, we were successful in performing HPD that involved both of these high-risk surgeries. Currently, the morbidity and mortality from HPD is still high at high-volume institutes [8]. Therefore, the prognosis for recovery and general health status are important factors in determining whether to perform invasive procedures.

The patient was diagnosed with advanced metastatic GB cancer at a previous hospital. Long survival is very rare for advanced GB cancer [9]. She could not receive adjuvant chemotherapy due to severe complications by S-1; however, she had no tumor relapse. CT imaging from a prior surgery showed that the cholangiocarcinoma was not remarkable. When the present disease was found, the tumor extending along the bile duct wall was suspicious for malignancy [10]. Since the tumor-free period was long, the surrounding infiltration was unclear. Following the evaluation of the papillary carcinoma by cholangioscopy, a new occurrence of cholangiocarcinoma was found. Based on the operative indication for EC, surgical planning was performed as described above.

In general, the nutritional status is low for these patients, and preoperative management for invasive surgery is usually required. However, our patient had very good nutritional status and was able to intake food normally. Thus, no special nutritional management was required.

For a surgeon in training, it is important to learn how to dissect postoperative adhesions. From our previous experience of 28 cases involving hepatectomy and pancreatectomy performed after abdominal surgery, half were performed by residents or fellowship surgeons. The surgical records and outcomes did not differ between instructors and trainees supported by experienced surgeons (in press). However, in this case, the first operation was poly-surgery with lymphadenectomy, and HPD requires more advanced techniques [11]. Therefore, experienced hepatobiliary pancreatic surgeons may perform the surgeries to reduce the operating time. We also tried to reduce the amount of intra-operative bleeding since blood loss also influences patient outcome [12]. However, in the region where the tumor, hepatic artery, and portal vein were tightly adhered together, the dissection was difficult and small amounts of bleeding were observed. Furthermore, accidental injury to the arterial branch in the remnant liver occurred during dissection. To mitigate these complications, plastic or vascular surgeons are usually placed on stand-by. In our case, an experienced plastic surgeon resolved this issue, and no severe adverse events after repair were observed. Furthermore, no blood transfusion was necessary, and the operating time was similar to that of a usual HPD at our institute. Based on our results, HPD surgeons should adopt an aggressive policy to treat patients who have undergone previous major abdominal surgeries.

The operative risk of HPD is higher than that of pancreaticoduodenectomy alone due to possible postoperative pancreatic fistula and related complications [13,14]. Furthermore, the first author experienced one mortality in 18 cases of HPD [15]. In our case, pancreatic fistula was not observed since we implemented Blumgart’s procedure for pancreatico-jejunostomy [16,17]. From our previous experiences of HPD, postoperative complications were not severe, only one patient undergoing major hepatectomy had transient hepatic failure, and there were no reports of death. Surgical site infections were observed in only 8%; therefore, the selected patients may not have been affected by severe surgical stress due to prior operations and adhesion as reported previously [18]. Furthermore, the hospitalization period did not increase. In our case, no infectious complications were observed, and the patient was discharged faster than expected.

## 4 Conclusion

We report a successful HPD for a female patient with extensive advanced EC who underwent previous poly-surgery for advanced GB carcinoma. Although there was arterial injury due to severe adhesion, it was resolved, and the patient was discharged uneventfully after surgery. Detailed information regarding tumor extension and liver function should be obtained preoperatively, and appropriate surgical simulation by three-dimensional imaging is important. This may increase operative indications for hepatobiliary cancer in order to achieve successful HPD with R0 resection and improved patient survival.

## Conflicts of interest

No COI.

## Funding

No funding.

## Ethical approval

Ethical permission for case report is obtained at our intuitional policy.

## Consent

Informed consent was obtained in this patient. Written informed consent was obtained from the patient for publication of this case report and accompanying images. A copy of the written consent is available for review by the Editor-in-Chief of this journal on request.

## Author contribution

All authors contributed the perioperative management and writing this paper. The first author, is a main operator and wrote this mainly.

ALL contributed patient operation and perioperative management equally in this case report. The first author was chairman and director of the department and a main operator. One co-author supported the arterial repair during operation. All authors approved the final version of the manuscript to be submitted.

## Registration of research studies

N/A.

## Guarantor

Professor Kunihide Nakamura, who is a cardiovascular surgeon, who is another chairman of our institute. Another guarantor is Associate professor, Hiroshi Ito, who is a last author, who is a plastic surgeon and contributed this operation.

## Submission declaration

The authors declare that the work described has not been published previously, that it is not under consideration for publication elsewhere, that its publication has been approved by all authors and either tacitly or explicitly by the responsible authorities where the work was carried out, and that, if accepted, it will not be published elsewhere—including electronically in the same form in English or any other language—without the written consent of the copyright holder.

## Provenance and peer review

Not commissioned, externally peer reviewed.
